# Tree pollen allergens—an update from a molecular perspective

**DOI:** 10.1111/all.12696

**Published:** 2015-08-06

**Authors:** C. Asam, H. Hofer, M. Wolf, L. Aglas, M. Wallner

**Affiliations:** ^1^ Department of Molecular BiologyUniversity of SalzburgSalzburgAustria

**Keywords:** allergen cross‐reactivity, allergen exposure, molecular allergology, tree pollen allergy, tree pollen sensitization

## Abstract

It is estimated that pollen allergies affect approximately 40% of allergic individuals. In general, tree pollen allergies are mainly elicited by allergenic trees belonging to the orders Fagales, Lamiales, Proteales, and Pinales. Over 25 years ago, the gene encoding the major birch pollen allergen Bet v 1 was the first such gene to be cloned and its product characterized. Since that time, 53 tree pollen allergens have been identified and acknowledged by the WHO/IUIS allergen nomenclature subcommittee. Molecule‐based profiling of allergic sensitization has helped to elucidate the immunological connections of allergen cross‐reactivity, whereas advances in biochemistry have revealed structural and functional aspects of allergenic proteins. In this review, we provide a comprehensive overview of the present knowledge of the molecular aspects of tree pollen allergens. We analyze the geographic distribution of allergenic trees, discuss factors pivotal for allergic sensitization, and describe the role of tree pollen panallergens. Novel allergenic tree species as well as tree pollen allergens are continually being identified, making research in this field highly competitive and instrumental for clinical applications.

Within the past century, allergic diseases have developed from being rather rare conditions into a pandemic health problem, and conservative estimates suggest that approximately half a billion people worldwide suffer from allergic rhinitis 1. In general, pollen allergens are considered a major risk factor for both seasonal allergic rhinitis and asthma, whereas indoor allergens appear to be a risk factor for perennial rhinitis. Still, some studies showed that more than 50% of patients with perennial allergic rhinitis are sensitized to pollen allergens 1, although sensitization profiles varied considerably depending on the geographic location of the study population. Pollen sensitization is usually restricted to anemophilous plants, which comprise approximately 10–18% of all flowering plants 2. To increase the chance of fertilization, wind‐pollinated plants have evolved characteristic strategies, including having small, dehydrated pollen with good aerodynamic properties that allow its dissemination over hundreds of kilometers 2, 3. Pollen grains initially enclose a single cell, which eventually develops into the male gametophyte. The inner pollen wall, called the intine, describes a typically multilayered thin cover composed of cellulose and pectin, whereas the exine refers to the very resistant outer wall, which provides robust protection of the pollen grain from disintegration. Apertures in the exine allow outgrowth of the pollen tube during pollination 4. Allergenic proteins are usually located within the pollen protoplast and readily released during the rehydration process. As was demonstrated for the birch pollen allergens Bet v 1 and Bet v 2, in the anhydrous state of the pollen, the allergens are located within the pollen cytoplasm, mostly in close proximity to ribosome‐rich areas. Upon rehydration, birch pollen allergens are released within minutes from apertures and subsequently found on the entire pollen surface 5, 6. Pollens of trees, grasses, and weeds have all been found to elicit allergic reactions in atopic individuals, but this review will focus on tree pollen allergens.

In general, trees are defined as woody perennial plants with a single or, in the case of coppicing, several self‐supporting main stems and a more or less defined crown. Some definitions also indicate a certain height or stem diameter as inclusion criteria to distinguish trees from shrubs 7. Allergenic trees may be found almost all over the world, reaching from the temperate climate zones of Europe, North America, and Asia, to the Mediterranean area, North Africa, parts of South America, South Africa, the Indian subcontinent, as well as parts of Australia, whereas in tropical climate regions tree pollen allergies are virtually absent (www.eol.org). Trees belonging to the orders Fagales, Lamiales, Proteales, and Pinales (www.allergen.org) (Fig. 1) are recognized as the most potent allergen sources, whereas in subtropical climates the Fabales trees mesquite (*Prosopsis juliflora*) and gulmohar (*Peltophorum pterocarpum*) have been acknowledged as clinically important allergen sources 8, 9. Moreover, date palms, which like grasses belong to the monocots, produce clinically relevant pollen allergens 10. Within the past decades, huge progress has been made in the identification and characterization of tree pollen allergens. The WHO/IUIS allergen nomenclature subcommittee maintains a systematic database of allergenic molecules. Allergens submitted to the database are reviewed by an executive committee. Therefore, in the present review, we focus on allergenic molecules acknowledged by the database, which comprises at present 53 tree pollen allergens from six botanical orders. A list of tree pollen allergens disclosing allergen families, functions, as well as the differentiation between major (in bold type) and minor allergens is provided in Fig. 2.

**Figure 1:**
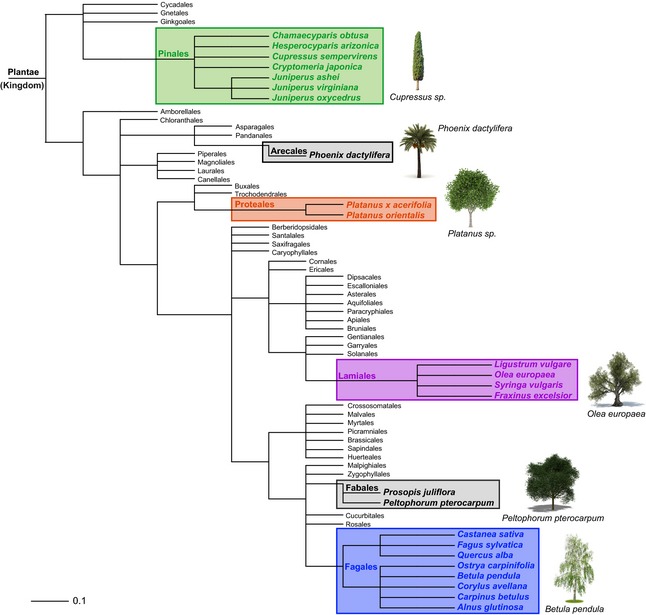
Phylogenetic tree of plant orders generated using the software phyloT a phylogenetic tree generator, based on NCBI taxonomy (http://phylot.biobyte.de/). Taxonomic orders containing species which have been acknowledged by the WHO/IUIS allergen nomenclature database (www.allergen.org) are highlighted in color (Pinales in green; Proteales in red; Lamiales in purple; Fagales in blue; and other in yellow). Photographs were obtained from Fotolia.

**Figure 2:**
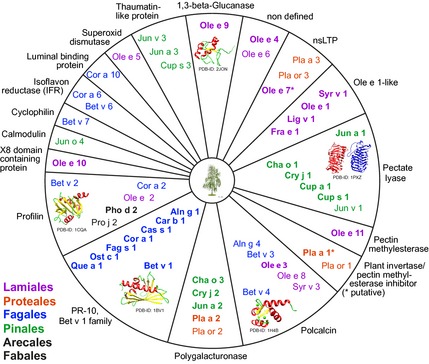
Schematic representation of tree pollen allergens clustered according to protein functions; major allergens are depicted in bold, minor allergens in regular font. Allergens of the Lamiales order are presented in purple, Proteales in red, Fagales in blue, Pinales in green, and other in black. Allergen structures were obtained from the RCSB PDB protein data bank (http://www.rcsb.org) and photographs from Fotolia.

## Fagales pollen allergens

**Figure 3:**
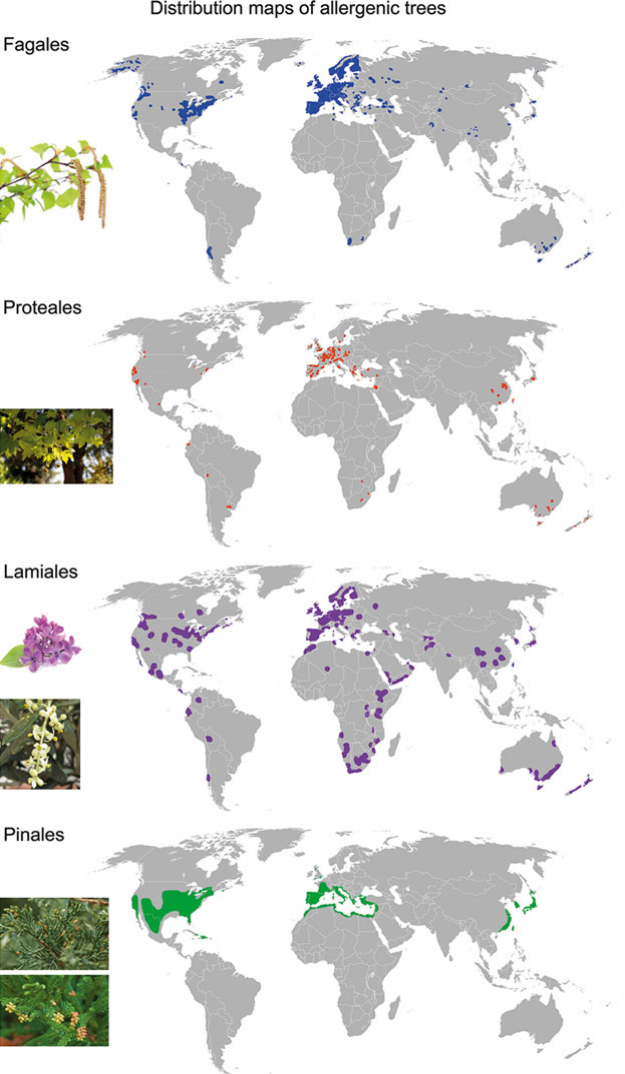
Geographic distribution of allergenic Fagales, Lamiales, Proteales, and Pinales species. The distribution data were extracted from maps provided on www.eol.org. Photographs of florescences of members of the Fagales (birch *Betula verrucosa*), Proteales (plane tree *Platanus acerifolia*), Lamiales (lilac *Syringa vulgaris* and olive *Olea europaea*), and Pinales (mountain cedar *Juniperus ashei* and Japanese cedar *Cryptomeria japonica*) were obtained from Fotolia.

Fagales tree pollens are the main cause of winter/spring pollinosis in the temperate climate zone of the Northern Hemisphere 11. The order Fagales consists of seven families, namely Betulaceae, Casuarinaceae, Fagaceae, Juglandaceae, Myricaceae, Nothofagaceae, and Ticodendraceae 12, although members of the Betulaceae (i.e. *Alnus*,* Betula*,* Carpinus*,* Corylus*, and *Ostrya*) and the Fagaceae (i.e. *Fagus*,* Castanea* and *Quercus*) are most frequently implicated in allergies. Moreover, pollens of the genera *Juglans*,* Myrica,* and *Casuarina* have been associated with tree pollen allergies (www.allergome.org). Most Fagales species show a tree or shrub‐like habitus with very small single‐sex flowers, which often grow as a dense‐flowered catkin‐like or spike florescence. Species of the order Fagales are distributed all over the globe, whereas almost all genera grow preferentially in temperate climate zones (Fig. 3).

### The Bet v 1‐like allergen family

The major Fagales pollen allergens Bet v 1 (birch), Aln g 1 (alder), Car b 1 (hornbeam), Ost c 1 (hop‐hornbeam), Cor a 1 (hazelnut), Fag s 1 (beech), Cas s 1 (chestnut), and Que a 1 (oak) belong to the pathogenesis‐related protein class 10 (PR‐10), which includes a large group of aeroallergens and common food allergens 13, 14. PR‐10 proteins are encoded by a diverse multigene family and share a small size of around 160 amino acids, a molecular mass of 17 kDa, a similar secondary structure, and are usually intracellular and cytosolic. Their 3D fold consists of three α‐helices embedded in an antiparallel β‐sheet consisting of 7 β‐strands 15. The core of the proteins is formed by an amphiphilic Y‐shaped cavity, which is solvent‐accessible via (in most cases) two to three openings on the surface 16. This structural motif may be the key feature of the biological function of PR‐10 proteins. Detailed crystallographic analysis of the major birch pollen allergen Bet v 1 using a wide spectrum of ligands revealed that the Bet v 1 binding pocket constitutes a promiscuous ligand‐complex binding site. Furthermore, different binding modes of Bet v 1 have been identified depending on different isoforms and the presence of other ligands 16. Recently, quercetin‐3‐O‐sophoroside (Q3OS), a glycosylated flavonol, was discovered to be a physiological ligand of Bet v 1. Flavonoids facilitate, among other things, pollen tube germination and are found to be stored as glycosylated precursors. Seutter von Loetzen et al. 17 speculate that, in the pollen, quercetin is glycosylated to Q3OS and stored as a Bet v 1‐Q3OS complex which protects the birch pollen DNA from UV damage, and then is released after pollen rehydration to be de‐glycosylated back into its active form. Over 90% of birch‐allergic individuals produce IgE antibodies against Bet v 1 and 60% of these atopic people react exclusively to the allergen 18, 19. Unexpectedly, a recent study revealed a resemblance of Bet v 1 to proteins of the lipocalin protein family, which most animal‐derived allergens belong to. The authors hypothesized that Bet v 1 is a carrier for iron–siderophore complexes and only shows its immunomodulatory capacity in its iron‐loaded state 20. Birch pollen extracts show a high degree of heterogeneity and contain a variety of Bet v 1 isoforms that differ by usually only few amino acids 21, 22. The homologous PR‐10 allergens within the Fagales show cross‐reactivity. Inhibition experiments revealed limited cross‐reactivity between allergens of the Betuloideae and Coryloideae families, thus suggesting that members of both families have the potential to sensitize susceptible individuals 23. As much as 50–93% of birch pollen‐allergic patients also develop allergic signs and symptoms against certain foods from the Rosid clade 24. The symptoms are manifested as a condition termed oral allergy syndrome and its occurrence typically involves presensitization to pollen allergens from the Fagales order 25.

### Minor Fagales pollen allergens

Besides Bet v 1, the minor birch pollen allergens Bet v 2, a profilin, Bet v 3 and Bet v 4, two members of the polcalcin family, the isoflavone reductase Bet v 6 (formerly named Bet v 5), and the cyclophilin Bet v 7 have all been identified as IgE‐binding proteins (the allergenic activity of profilins and polcalcins is discussed below). Bet v 6 produces sensitization rates of 32% among birch pollen‐allergic patients with CAP classes >3 26. Moreover, the homologous allergen (Cor a 6) has been identified in hazel pollen, showing the typical molecular weight of 34 kDa. In an ELISA experiment, 14% of hazel and birch pollen‐allergic patients reacted to recombinant Cor a 6 (www.allergen.org). The chaperone Cor a 10, a 70‐kDa luminal binding protein of the 70‐kDa heat‐shock protein family, was identified in hazel pollen and showed cross‐reactivity with 70‐kDa proteins from other tissues and species 27. Bet v 7, a member of the cyclophilin A family, is a protein which is induced after stresses such as wounding and chemical exposure. No immunological cross‐reactivity of plant and nonplant cyclophilin A family members has been reported; however, rabbit IgG raised against recombinant Bet v 7 could cross‐react with other plant‐derived cyclophilins 28. Very recently, birch pollen glutathione‐S‐transferase (GST) was identified as a novel IgE‐binding protein, showing sensitization rates of 13% within a cohort of birch pollen‐allergic patients. Despite the fact that GST is abundant in birch pollen, the quantities released are lower than for Bet v 1. Thus, the authors speculate that the low IgE‐binding frequency could be linked to lower exposure to this novel pollen allergen 29.

## Allergenic molecules identified with Proteales pollen

The order of Platanaceae, previously indexed within the Hamamelidales, was recently reclassified as a member of the order Proteales 12. Of the four families comprising the Proteales, allergenic species are exclusively found within the Platanaceae. Platanaceae are ‘living fossils’ which extend back to the Early Cretaceous, and the members of the genus *Platanus* frequently hybridize, forming new species 30. These monoecious trees are deciduous and grow sympodially, and the flowers are reduced and show ball‐like structures. *Platanus* species, usually called plane trees, although some North American species are referred to as sycamores, are important sources of airborne allergens. They grow preferentially in northern temperate regions (Fig. 3). Due to their resistance against diseases and air pollution, they are widely used as ornamental trees in the United States and Western Europe. To date, only the species *Platanus acerifolia* (London plane tree) and *Platanus orientalis* (oriental plane) are recognized as allergenic trees by the WHO/IUIS.

*Platanus acerifolia* is a hybrid originated from *Platanus occidentalis* and is often planted in cities of North America, Australia and New Zealand, South Africa, and Europe. During its flowering season, the released pollen can attain high levels, for example, up to 14% of total pollen in some areas of Spain 31. Three different pollen allergens have been described for *Platanus acerifolia*. The nonglycosylated 18‐kDa Pla a 1 is a putative plant invertase inhibitor. It is a major allergen, although its relative amount in extracts represents less than 0.5% of the whole protein content 31. In mono‐sensitized and poly‐sensitized *Platanus*‐allergic patients in Spain, up to 92% and 83%, respectively, recognize Pla a 1, which is responsible for about 60% of the total IgE‐binding capacity of plane tree pollen extract 31, 32. Pla a 2, the second major allergen, is a 43‐kDa glycoprotein belonging to the polygalacturonase family. The functional enzyme shows homology to other pollen and food polygalacturonase allergens. More than 84% of patients allergic to plane tree react to Pla a 2, which represents about 52% of the total IgE‐binding capacity of plane tree pollen extracts 33. The 10‐kDa nonspecific lipid transfer protein (nsLTP) Pla a 3 makes up 0.08% of total pollen protein and shows 58.3% sequence identity with nsLTP Pru p 3, the major allergen from peach. Pla a 3 is recognized by 27.3% of plane pollen‐allergic patients; however, reactivities of up to 63.8% have been reported among Pru p 3‐allergic patients in Mediterranean areas. Interestingly, 16.6% of patients without sensitization to Pru p 3 recognize Pla a 3, indicating species‐specific IgE epitopes 34.

*Platanus orientalis* is mainly present in southwest Asia, southeast Europe, but also in Arabic countries such as Iran. During its flowering season in early spring, pollen concentrations can peak to 15% of total pollen counts. In a study including 19 Iranian *Platanus orientalis*‐allergic patients, the 18‐kDa plant invertase/pectin methylesterase inhibitor Pla or 1, the 42‐kDa polygalacturonase Pla or 2, and the 12‐kDa nsLTP Pla or 3 with IgE‐binding prevalences of 21%, 27%, and 27%, respectively, were identified. The allergens of Platanus orientalis show great similarities to the three main allergens of *Platanus acerifolia* 35.

## Lamiales pollen allergens

Within the order Lamiales, only the Oleacea family contains allergenic wind‐pollinated tree species. Oleaceae were formerly classified within the order Scrophulariales; however, recent phylogenetic studies led to the combining of Lamiales and Scrophulariales to form the order Lamiales 12. Allergenic Lamiales species are endemic in vast parts of Europe but are also scattered over North America, Africa, Asia, and Australia (Fig. 2). Within the Oleaceae, pollens of four tree species have been acknowledged by the WHO/IUIS subcommittee to contain allergenic proteins, which are European ash (*Fraxinus excelsior)*, common privet (*Ligustrum vulgare*), olive (*Olea europea*), and lilac (*Syringa vulgaris*). Of note, all four species contain a member of the highly cross‐reactive IgE‐binding Ole e 1‐like protein family. The allergens show high sequence identities of more than 80% but have differences in glycosylation patterns 36, 37, 38.

### Ole e 1—the major IgE‐binding component of olive pollen

In the Mediterranean area, due to large tracts of cultivated olive trees, the most prevalent sensitizer for Lamiales pollen allergies is olive pollen. The sensitization rates among patients with respiratory symptoms may reach levels of 70% in some areas of Italy, and in a Spanish cohort of pollinosis patients, 75.3% reacted with the major olive pollen allergen Ole e 1 39, 40. Depending on latitude as well as geographic location, the flowering period of olive trees can vary from January to mid‐July in some areas in Northern Italy, and pollination can be either entomophilous or anemophilous, depending on the level of pollen production 39. Nevertheless, with a sensitization prevalence of more than 80% among olive pollen‐allergic patients, Ole e 1 represents the major IgE‐binding component of olive pollen. This may be a consequence of the high abundance of the allergen, which accounts for approx. 20% of the total protein in pollen extracts 41. The biological function of Ole e 1 has not yet been elucidated, but the allergen is thought to be involved in pollen hydration and germination processes 42. Structurally, Ole e 1 is a glycoprotein with a backbone of 145 amino acids corresponding to a size of approx. 20 kDa; however, a minority of the protein also appears nonglycosylated and migrates at 18.5 kDa in SDS‐PAGE. The N‐glycosylation site is located at residue Asn111 41, 43. According to circular dichroism analyses, Ole e 1 is composed of 22% α‐helices, 38% β‐structures, and 40% turns or random conformations, and both glycosylation and an intact structure seem crucial for antibody recognition 41, 44. Due to either similar folding and/or glycosylation pattern, Ole e 1 shows cross‐reactivity not only with Ole e 1‐like protein from allergenic Oleacea species, but also to a lower degree with other members of the Lamiales (i.e. *Plantago lanceolata*), Caryophyllales (i.e. *Chenopodium album*), or Poales (i.e. *Lollium perenne* or *Phleum pratense*) 42, 45.

### The spectrum of olive pollen allergens

Besides Ole e 1, 10 other olive pollen allergens have been entered into the IUIS database (www.allergen.org). The profilin Ole e 2 (15 kDa) and the polcalcin Ole e 3 (9 kDa) belong to the panallergens. Little is known about Ole e 4 (32 kDa), but the allergen shows similarities to the N‐terminal domain of Ole e 9, suggesting that Ole e 4 might be a degradation product of this allergen 45. Ole e 5, a 16‐kDa protein, belongs to the family of Cu/Zn super‐oxide dismutases (SODs) and shows homology to other plant‐specific SODs. The enzymatic activity of Ole e 5 has been confirmed. Of note, the allergen is not solely expressed in pollen, but also in other plant tissues. About 39% of olive‐sensitized patients recognize Ole e 5 45. With only 50 amino acids in its sequence, Ole e 6 (10 kDa) is a rather small cysteine‐enriched allergen, but up to 50% of olive pollen‐allergic patients are sensitized to the protein 41. Interestingly, the expression of Ole e 6, 7, 9, 10, and 11 in pollen seems highly variable and dependent on the geographic location of the plants 45. Olive pollen also contains a nonspecific lipid transfer protein (ns‐LPT), Ole e 7 (10 kDa), which belongs to the PR‐14 family. In general, the protein is a scarce allergen, with sensitization rates below 10% among olive pollen‐allergic individuals; however, in areas where pollen counts exceed 5000 counts/m^3^, sensitization rates are as high as 60% 42. IgE antibodies against Ole e 7 have been associated with increased risk of food anaphylaxis, and patients sensitized to either Ole e 7 or Ole e 9 experienced more side‐effects during immunotherapy. Thus, sensitivity to either allergen may indicate greater severity of the disease. Of note, in some areas with extremely high olive pollen exposure, some patients have IgE against Ole e 7 but not Ole e 1 40. The allergen Ole e 8 is a 4 EF‐hand calcium‐binding protein with a molecular weight of 20 kDa, having a biological role in signal transduction. In prickly juniper, the allergen Jun o 4 is also a 4 EF‐hand polcalcin; nevertheless, the homology with Ole e 8 is restricted to the EF‐hand motifs 45. The beta‐glucanase Ole e 9 belongs to the PR‐2 allergen family and consists of two domains. The C‐terminal domain has a carbohydrate‐binding moiety and shows homology to Ole e 10, whereas the N‐terminal domain contains the active site of the enzyme 41. Sensitization rates of up to 35% have been reported among olive pollen‐allergic patients 40. The allergen is expressed at very low levels in olive pollen, and thus, there is considerable variability of Ole e 9 content in commercial extracts 46. Even though Ole e 10 is present only in small amounts in pollen, it is considered a major allergen, causing sensitization rates of 55% in olive pollen‐allergic patients. The protein was shown to bind 1,3‐beta‐glucans and has been detected in the outgrowing pollen tube in close proximity to callose molecules, suggesting a role in pollen wall re‐formation. Moreover, Ole e 10‐specific IgE binding can be inhibited with various pollen, vegetable, and latex extracts 41. The pectin methylesterase Ole e 11 completes the panel of olive pollen allergens. The 37.4 kDa protein provokes sensitization rates of up to 75% and shows homology to Sal k 1, the major allergen from Russian thistles 45. Of note, polygalacturonases, pectin methylesterases, and pectate lyases are typically found in plant pathogenic bacteria or fungi; however, many major pollen allergens as well belong to these families of pectin‐degrading enzymes, a fact that deserves some research attention.

### Allergens identified in the pollen of ash and lilac

Although only one allergen from European ash, Fra e 1, has been reported by the IUIS, the plant is recognized as potent allergen source. Ash trees are endemic in deciduous forests mainly in North America, Europe, and parts of Asia, but can also be found in small numbers in some areas of the Southern Hemisphere. In Europe ash, pollen counts can reach similar levels as birch, and also the flowering seasons of both trees show considerable overlap. In Austria, a sensitization rate to ash pollen of 17.6% has been reported among pollen‐allergic individuals, whereas ash pollen was responsible for 4% sensitization of allergic patients from Strasbourg 37, 47. The major ash pollen allergen is a glycoprotein, belongs to the Ole e 1‐like protein family, and exhibits 82%, 88%, and 91% amino acid sequence identity with lilac Syr v 1, olive Ole e 1, and privet Lig v 1, respectively 37. Between 75% and 86% of ash pollen‐allergic individuals show IgE reactivity to Fra e 1 37, 48. In a study by Palomares et al., IgE binding to olive and ash pollen extracts was analyzed by comparing sera of olive pollen‐exposed patients from Spain with Austrian ash‐exposed patients. The authors found substantial cross‐reactivity between the two Ole e 1‐like proteins 49.

Lilac (*Syringa vulgaris*) and common privet (*Ligustrum vulgare*) are two members of the Lamiales which have both been implicated in allergies and asthma. Both plants are endemic in Europe where they are increasingly cultivated for ornamental purposes, but can also be found in other parts of the world such as Asia or North America. Interestingly, privet is an insect‐pollinated tree; thus, environmental pollen concentrations are usually very low; nevertheless, there is evidence that privet can act as sensitizer for Lamiales allergies 50. The major allergens of lilac and privet are Syr v 1 and Lig v 1, respectively, which are members of the Ole e 1‐like protein family. Extensive cross‐reactivity between Ole e 1, Syr v 1, and Lig v 1 has been demonstrated 38. Besides group 1 allergens, Syr v 3, a 2 EF‐hand polcalcin, has been identified in lilac pollen 51.

## Allergenic molecules identified within the order Pinales

In contrast to Fagales and Lamiales, the Pinales, formerly known as Coniferales, are gymnosperms, indicating that their seeds are not covered by a carpel. The Pinales comprise seven families: Araucariaceae, Cephalotaxaceae, Pinaceae, Podocarpaceae, Sciadopityaceae, Taxaceae, and Cupressaceae; however, only certain genera of the Cupressaceae (*Chamecyparis*,* Cryptomeria*,* Cupressus*,* Hesperocyparis*, and *Juniperus*) have been acknowledged by the IUIS to contain clinically relevant allergens (www.allergen.org). In general, these robust trees are distributed all over the Northern Hemisphere, predominant in arctic and alpine regions, whereas allergenic Pinales species are found predominantly in warmer climates (Fig. 3) 52, 53, 54. In the Mediterranean area as well as in southern USA, *Cupressus sempervirens* (Mediterranean cypress) and *Cupressus arizonica* (Arizona cypress) are significant sources of pollen allergens, causing sensitization rates from 2.4% to 35.4% in the general population 55 and affecting up to 42.7% of allergic individuals in Italy 56. Their major allergens Cup a 1 57 and Cup s 1 are highly related, sharing 95.1% sequence identity, and have been identified as allergenic pectate lyases. *Cryptomeria japonica* (Japanese cedar or Sugi) as well as *Chamaecyparis obtusa* (Japanese cypress) are endemic to the Japanese islands, Taiwan, and parts of neighboring Chinese coastal areas. Their pollen are a major cause of seasonal rhinitis in Japan, affecting up to 40% of the population in certain age groups and areas 58, 59. Their two major allergens Cry j 1 60 and Cha o 1 61 share 78.6% sequence identity, while the homology of Cry j 1 with Cup a 1 is 79% 62.

### Tree pollen allergens belonging to the pectate lyase family

The allergens Cry j 1 and Cup a 1 belong to the pectate lyase protein family and show high levels of cross‐reactivity 62. Moreover, pectate lyase allergens have been identified in the pollen of the juniper species *Juniperus ashai* (mountain cedar Jun a 1), a plant which is native to southern and eastern parts of the USA, and *Juniperus virginiana* (eastern red cedar Jun v 1) 63, 64. However, the latter is of minor importance for allergic individuals 64. Very recently, we conducted a study to compare IgE cross‐reactivity of different cohorts (Italy, Japan) to the purified natural pectate lyases Jun a 1, Cup a 1, and Cry j 1. In inhibition experiments with Italian patients' sera, we found that the highly homologous allergens Cup a 1/ Jun a 1 inhibited binding to themselves to approx. 80–90%, whereas Cry j 1 inhibited IgE binding to Cup a 1 by 52%. IgE binding to coated Cry j 1 was fully inhibited by all three allergens. In Japanese patients, the Cup a 1/ Jun a 1 group inhibited binding to themselves to approx. 60–80%, to coupled Cry j 1 to 44%, whereas Cry j 1 inhibited binding to itself by 90%. Of note, the Cup a 1/ Jun a 1 group hardly inhibited IgE binding to coated Cry j 1 (only up to 15%). This indicates that patients show an exclusive IgE epitope pattern to the pectate lyase allergen they are actually exposed to, with only limited cross‐reactivity between the allergen clusters 65.

Pectate lyase allergens have been identified as major allergens not only in the pollen of Cupressacea trees, but also in Asteraceae weeds. The allergen family comprises proteins with a molecular weight of approx. 37 kDa. The typical central beta‐helix structure of allergenic pectate lyases consists of three parallel beta‐sheets. The core unit is surrounded by several short alpha‐helices and beta‐strands, which form the active center of these enzymes 66. After providing evidence that Cry j 1 is an active enzyme, it has been suggested that the pectolytic activity, which is generally necessary for plant tissue remodeling, might play a role in pollen tube outgrowth 67. Unlike the nonglycosylated allergenic pectate lyases from weed pollen, tree pollen pectate lyase allergens contain N‐linked glycosyl structures. The first glycostructure was determined for Cry j 1, followed by reports of the glycosylation patterns of Jun a 1 and Cup a 1 68, 69, 70. Upon determination of the glycostructures attached to Cha o 1, Kimura et al. proposed that all Cupressaceae pectate lyase pollen allergens share at least one common glycosyl structure 71, and thus, such motifs may also mediate the binding of cross‐reactive carbohydrate determinant‐specific IgE antibodies responsible for a variety of clinically irrelevant positive IgE tests. For Cup a 1, it was reported that periodate‐treated allergen extracts almost completely lost IgE binding 72, and also nonglycosylated recombinant Cup a 1 produced in *E. coli* showed much lower IgE reactivity than the natural allergen or Cup a 1 produced as a glycoprotein in reticulocytes 57.

### Further Pinales pollen allergens

Besides pectate lyases, the polygalacturonases, Cha o 2, Cry j 2, and Jun a 2 have been identified as major allergens in Pinales pollen. These 43‐kDa allergens show sequence identities between 71% and 82% and also share cross‐reactive IgE epitopes. Sensitization rates of up to 82.5% among Cupressaceae‐allergic patients have been reported for group 2 allergens, rendering these proteins major allergens of Pinales pollen 73, 74. In juniper and cypress pollen, the thaumatin‐like allergens Jun a 3, Jun v 3, and Cup s 3 have been described as minor allergens. These allergens, belonging to the PR‐5 family, are recognized by approx. 33–47% of juniper‐ or cypress‐allergic patients 75, 76. Ultimately, a unique allergen was described in *Juniperus oxicedrus*, termed Jun o 4, which is a 4 EF‐hand calcium‐binding protein (www.allergen.org).

## Tree pollen panallergens

Panallergens are generally recognized as ubiquitously expressed allergenic proteins. The most important tree pollen panallergens belong to the profilin and polcalcin families, although Bet v 1‐related proteins as well could in principle be classified as panallergens. To date, five tree pollen profilins and six tree pollen polcalcins have been identified (Fig. 2). In general, profilins are cytosolic proteins present in all eukaryotic cells. Despite their highly variable protein sequence, profilins show a much conserved structure consisting of a compact beta‐sheet forming the core unit surrounded by several alpha‐helices 77. Profilins are actin‐binding proteins; however, they can also bind other ligands such as poly‐L‐proline or phosphoinositide. The latter are implicated in many essential pathways ranging from cell growth to cell death, including vesicular transport, regulation of ion channels, and modulation of lipid metabolism 78. Thus, roles for profilins in cellular processes such as endo‐ and exocytosis, signal transduction, actin polymerization, cell mobility, cell division, and pollen tube outgrowth have been suggested 79. Polcalcins represent the second group of panallergens found in tree pollen. The exact function(s) of polcalcins remains elusive; however, due to their pollen‐specific expression, roles in the regulation of intracellular calcium levels as well as in pollen germination have been suggested 79. Polcalcins share common calcium‐binding EF‐hand motifs and, according to the number of EF‐hands, tree polcalcins have been clustered in 2 (Bet v 4, Aln g 4, Fra e 3, Ole e 3, Syr v 3), 3 (Bet v 3), and 4 (Jun o 4, Ole e 8) EF‐hand proteins. The structure of polcalcins is mainly alpha‐helical, and cross‐reactivity with other polcalcins is frequently reported 80. The clinical importance of profilins and polcalcins has been debated extensively. In general, the sensitization rates to profilins and to polcalcins among tree pollen‐allergic patients are rather low and heavily influenced by exposure levels, geographic factors, and patient ages 81, thus making it extremely difficult to formulate general statements. Analyses of sensitization rates toward Bet v 2 revealed 5–7% reactivity using sera from birch pollen‐allergic individuals from Northern European countries, whereas patients from Central and Southern Europe showed sensitization rates of up to 45% 82. In a recent study using sera from Middle‐European allergic patients, Bet v 2 sensitization was 9.4% and sensitization to Bet v 4 4.2% 83. Among olive pollen‐allergic patients, sensitization rates toward the profilin Ole e 2 are around 24%, and IgE prevalence to the polcalcin Ole e 3 is between 20 and 30% 84. Both profilins and polcalcins are vastly cross‐reactive across species 77, 79; thus, patients sensitized to either of these panallergens may show multiple sensitizations toward biologically unrelated sources—a problem frequently associated with extract‐based diagnosis. As mentioned, the clinical relevance of these panallergens is limited and usually restricted to food profilins rather than pollen allergens. However, in some pollens (e.g. date palm), profilin has been shown to be a major allergen. Unlike trees, palms are monocots. Similar to tree ferns, palm trees do not show secondary growth of their stems; nevertheless, their tree‐like habitus legitimates their mentioning in this review. Sensitization rates to date palm pollen among patients with allergic respiratory symptoms range from 19% in Spain to 25% in Saudi Arabia, whereas the profilin Pho d 2 is recognized by 56% of the same patients 10. Of note, several other IgE‐reactive proteins from atopic sera have been identified showing IgE‐binding rates of > 50%; however, they have not been entered into the IUIS database so far 85.

## Novel allergenic trees and tree pollen allergens

Besides the many officially acknowledged tree pollen allergens, companies offer a wide variety of extracts for allergy diagnosis, including extracts from many tree species not listed in the IUIS database. Especially in the arid and subtropical regions of the world, tree pollinosis represents an increasing health problem. Mesquite trees (*Prosopis juliflora*), belonging to the order Fabales, have been acknowledged as potent elicitors of respiratory allergies in India, North America, and the Arabian Peninsula. So far, only the profilin Pro j 2 has been identified and characterized. Nevertheless, mesquite pollen extracts contain multiple IgE‐reactive proteins ranging from 14 to 95 kDa, suggesting that many more allergens will be identified within the next years 8. Acacia (wattle) trees, also belonging to the same order as mesquite, have been reported to elicit sensitization rates of up to 48% in pollinosis patients in Arab countries. Inhibition studies with pollen extracts suggest a high level of cross‐reactivity between wattle and mesquite pollen allergens, but also between wattle and grass allergens 86. The pollen of *Peltophorum pterocarpum* (yellow gulmohar tree), which contains eight IgE‐reactive protein components, was shown to be responsible for the nearly 33% sensitization rate in respiratory allergic patients from Calcutta 9. *Ricinus communis* (castor bean) is an oilseed crop plant growing in warm regions of the world and represents the source of castor oil. So far, the 2S albumins Ric c 1 and Ric c 3 have been isolated from castor bean seeds, but also the pollen of this anemophilous plant have been implicated in rhinitis and/or asthma. In a study carried out in Malaga, Spain, sensitization rates of almost 8% to *Ricinus* pollen were reported 87. Also, in temperate climate zones, there are allergenic trees which have not been extensively characterized so far. In a study of 371 allergic patients from the New York area, significant sensitization rates to oak (34.3%), birch (32.9%), maple (32.8%), beech (29.6%), hickory (27.1%), ash (26%), elm (24.6%), and poplar (20.6%) tree pollen extracts suggest clinical importance of all of these tree pollens 88, whereas a study of Swedish patients indicates that aspen, linden, elm, sallow, maple, and poplar pollen are clinically irrelevant allergen sources 89. In an allergic population from the area of Ankara, Turkey, 42.3% of the patients had positive skin prick tests to poplar, 46% to salix, but less than 40% reacted positive with Fagales extracts, and pine elicited positive skin reactions in 24% of the subjects 90. Yet another study suggests an important contribution to aero‐allergen‐associated symptoms by the pollens of acer and salix 91.

## Summary and concluding remarks

Advances in molecular allergology paved the way for molecule‐based allergy diagnosis beyond the botanical identification of allergenic trees. This was a milestone for our understanding of tree pollen allergens and the complex interplay of allergic sensitization and cross‐reactivity. Nevertheless, much research needs to be carried out to complete the panel of tree pollen allergens. In general, sensitization prevalence seems to be highly dependent on the pollen exposure pattern of a given population. In our present understanding, tree pollen allergies are mainly a problem of industrialized societies within the temperate climate zones, where the most potent allergenic trees are endemic. However, along with the identification of new allergenic tree species, it is becoming more and more evident that tree pollen allergies may become an increasing problem for societies within subtropical climate zones. In our opinion, this emerging health problem will require increasing attention and research. Thus, it might be necessary to revise our view about the importance of allergenic tree pollen species, which will eventually also induce changes in the field of allergy diagnosis and therapy.

## Author contributions

All authors contributed in collecting information for the review and in writing the review. CA and MW performed the final editing.

## Conflicts of interest

All authors declare no conflict of interest.
